# Cellular mechanisms underlying *Pax3-*related neural tube defects and their prevention by folic acid

**DOI:** 10.1242/dmm.042234

**Published:** 2019-11-22

**Authors:** Sonia Sudiwala, Alexandra Palmer, Valentina Massa, Alan J. Burns, Louisa P. E. Dunlevy, Sandra C. P. de Castro, Dawn Savery, Kit-Yi Leung, Andrew J. Copp, Nicholas D. E. Greene

**Affiliations:** Developmental Biology and Cancer Research and Teaching Department, UCL Great Ormond Street Institute of Child Health, University College London, London WC1N 1EH, UK

**Keywords:** Folic acid, Neural tube defects, Pax3, Cell cycle

## Abstract

Neural tube defects (NTDs), including spina bifida and anencephaly, are among the most common birth defects worldwide, but their underlying genetic and cellular causes are not well understood. Some NTDs are preventable by supplemental folic acid. However, despite widespread use of folic acid supplements and implementation of food fortification in many countries, the protective mechanism is unclear. *Pax3* mutant (*splotch*; *Sp^2H^*) mice provide a model in which NTDs are preventable by folic acid and exacerbated by maternal folate deficiency. Here, we found that cell proliferation was diminished in the dorsal neuroepithelium of mutant embryos, corresponding to the region of abolished Pax3 function. This was accompanied by premature neuronal differentiation in the prospective midbrain. Contrary to previous reports, we did not find evidence that increased apoptosis could underlie failed neural tube closure in *Pax3* mutant embryos, nor that inhibition of apoptosis could prevent NTDs. These findings suggest that Pax3 functions to maintain the neuroepithelium in a proliferative, undifferentiated state, allowing neurulation to proceed. NTDs in *Pax3* mutants were not associated with abnormal abundance of specific folates and were not prevented by formate, a one-carbon donor to folate metabolism. Supplemental folic acid restored proliferation in the cranial neuroepithelium. This effect was mediated by enhanced progression of the cell cycle from S to G2 phase, specifically in the *Pax3* mutant dorsal neuroepithelium. We propose that the cell-cycle-promoting effect of folic acid compensates for the loss of Pax3 and thereby prevents cranial NTDs.

## Abstract (precis)

**Summary:** Neural tube defects in a folic-acid-responsive, folate-sensitive mouse model are associated with a localised proliferation defect in the neuroepithelium. Supplemental folic acid stimulates progression through S phase to correct this abnormality.

## INTRODUCTION

Neural tube defects (NTDs) such as anencephaly and spina bifida are among the most common birth defects worldwide, affecting more than 250,000 pregnancies every year (Copp et al., 2013; Zaganjor et al., 2016). Maternal use of supplemental folic acid (FA) prevents a proportion of NTDs (Berry et al., 1999; Czeizel and Dudás, 1992; MRC Vitamin Study Research Group, 1991), and FA food fortification programmes, which now exist in many countries, have generally been associated with a reduction in NTD prevalence, compared with historical pre-fortification frequencies (Berry et al., 2010; Castillo-Lancellotti et al., 2013). A key aim for public health efforts is now to ensure that women who might become pregnant achieve recommended daily intakes of FA, in order to lower NTD rates (Martinez et al., 2018). However, there is still a gap in our knowledge concerning the mechanism by which FA prevents NTDs in the developing embryo. FA is the synthetic form of folate, a term that refers to a group of molecules, based on a tetrahydrofolate (THF) backbone, that carry one-carbon (1C) units in folate 1C metabolism. FA is converted via dihydrofolate to THF, with subsequent addition of 1C groups derived principally from serine, glycine and formate (Tibbetts and Appling, 2010; Leung et al., 2017).

NTDs result from incomplete closure of the neural tube during embryonic development (Greene and Copp, 2014; Nikolopoulou et al., 2017). Among mouse genetic models, exencephaly and/or spina bifida arise in *splotch* mice, carrying mutations of the paired-box-domain-containing transcription factor Pax3 (Epstein et al., 1991; Greene et al., 2009). Notably, *Pax3* mutants (*Pax3^Sp2H^*, *Pax3^Sp^*) are among the very few models in which NTDs have been found to be both preventable by supplemental FA and exacerbated by maternal folate deficiency, imposed by diet and administration of antibiotic to remove folate-synthesizing gut bacteria (Burren et al., 2008; Fleming and Copp, 1998; Wlodarczyk et al., 2006). Hence, NTDs in this model are both FA responsive and folate sensitive. In addition to mutation of the *Pax3* gene itself, suppression of *Pax3* expression in mouse embryos is also proposed to contribute to NTDs induced by environmental factors, such as maternal diabetes (Fine et al., 1999; Machado et al., 2001) and polycyclic aromatic hydrocarbons (Lin et al., 2019).

Mutations of the human *PAX3* coding sequence have been identified in some individuals with NTDs (Hart and Miriyala, 2017) and may contribute to a minority of NTDs. Altered methylation of *PAX3* has also been identified in NTD cases, suggesting that altered expression could potentially play a contributory role (Lin et al., 2019).

Understanding the mechanisms by which *Pax3* loss of function prevents neural tube closure will not only give insight into possible causes of NTDs but could also provide an opportunity to better understand the means by which FA prevents NTDs. It has been proposed that *Pax3*-related NTDs (specifically the *Pax3^Sp^* allele) result from excess apoptosis: NTDs were prevented by genetic or pharmacological suppression of p53 function (Pani et al., 2002), leading to the hypothesis that Pax3 functions to suppress p53-dependent apoptosis in the neuroepithelium. A p53-dependent excess of apoptosis has also recently been proposed to underlie NTDs associated with zinc deficiency (Li et al., 2018). Both excess and insufficient apoptosis have been associated with exencephaly in other mouse mutants, although – in most cases – a causal relationship has not been definitively proven (Greene and Copp, 2014; Nikolopoulou et al., 2017).

Other studies of apoptosis in *splotch* (*Pax3^Sp^*) embryos have produced differing findings. For example, increased TdT-mediated dUTP-biotin nick-end labelling (TUNEL) staining was reported in the neural tube at embryonic day (E) 10.5 (Pani et al., 2002), whereas no change in the number of pyknotic nuclei in the spinal neuroepithelium was detected at E9.5 (Kapron-Brás and Trasler, 1988). Moreover, although aberrant levels of apoptosis have been demonstrated in *Pax3^Sp/Sp^* and *Pax3^Sp2H/Sp2H^* mutants in the dermomyotome of the developing somites, increased apoptosis was not observed in the neural tube at E9.5 or later stages (Borycki et al., 1999; Mansouri et al., 2001). In the current study, we sought to address the question of the possible contributory role of apoptosis to NTDs in the *Pax3^Sp2H/Sp2H^* model and to investigate other potential causative cellular abnormalities. Having identified a tissue-specific defect in cellular proliferation, we went on to ask whether this abnormality was corrected by FA supplementation in association with prevention of NTDs.

## RESULTS

### NTDs in *Pax3* (*Sp^2H^*) mutant embryos do not result from excess apoptosis

NTDs in *splotch* embryos result from a cell-autonomous defect in the neuroepithelium (Goulding et al., 1991; Li et al., 1999). Therefore, if excess apoptosis is the cause of cranial NTDs in *Pax3* mutants, this should be detectable prior to and/or during closure of the cranial neuroepithelium, which has not previously been examined.

The initiation of neural tube closure, at the hindbrain-cervical boundary (Closure 1; five to six somites; E8.5) and in the posterior forebrain (Closure 2; nine to ten somites; E9.0), occurs similarly in *Pax3^Sp2H/Sp2H^* embryos and wild-type littermates. However, progression of ‘zippering’ forwards from Closure 1 and backwards from Closure 2 fails in those mutants that develop midbrain/hindbrain exencephaly (Fleming and Copp, 2000). In the current study, exencephaly, characterised by persistently open cranial neural folds, arose in 65% of *Pax3^Sp2H/Sp2H^* mutants (*n*=46), whereas all *Pax3^+/+^* embryos (*n*=54) completed closure by the 16-somite stage at E9.5 (*P*<0.001; chi-square test).

**Fig. 1.**
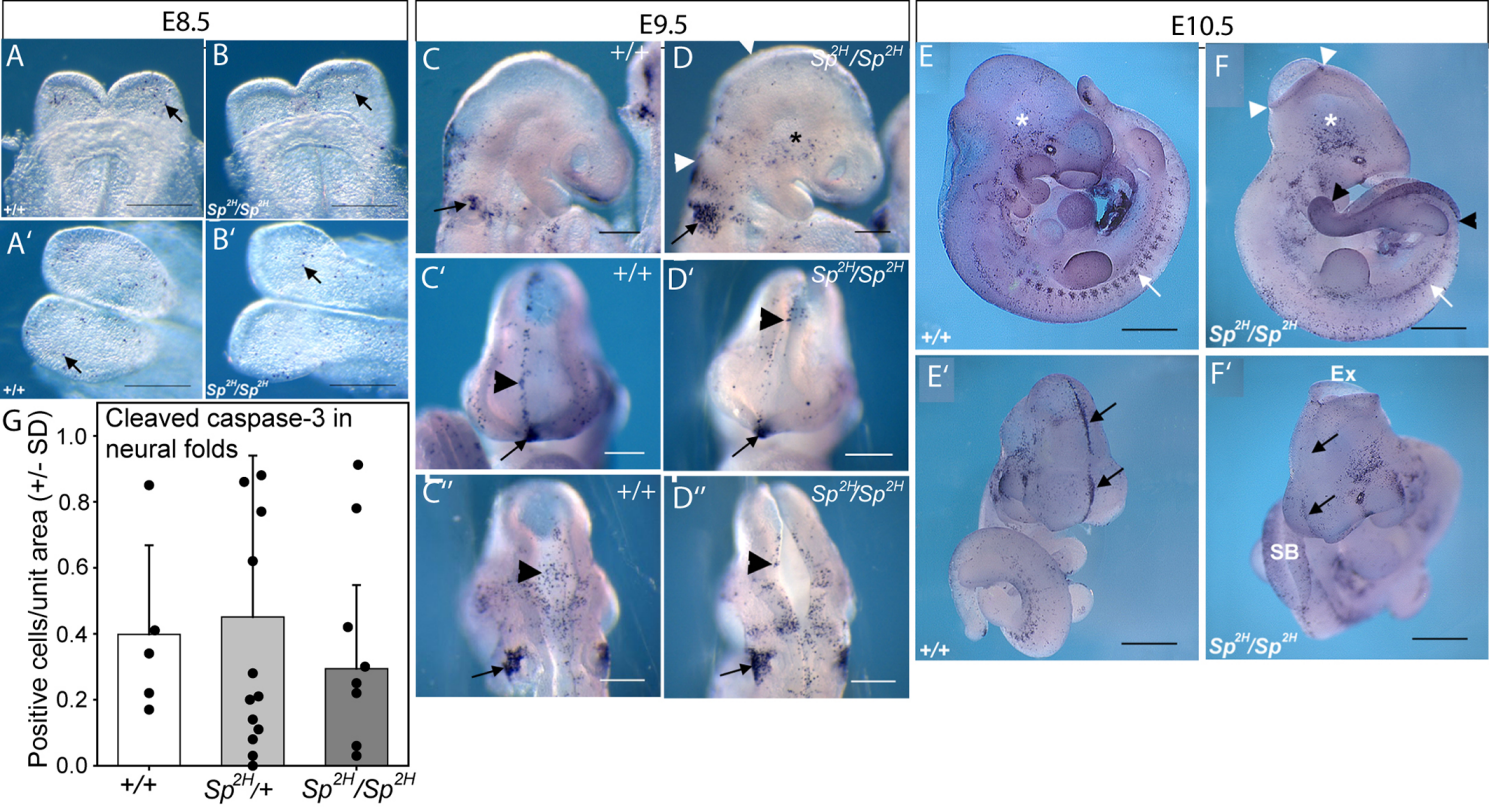
**Apoptosis in the neuroepithelium is not affected by *Pax3* genotype.** (A-F) TUNEL staining of embryos at E8.5 (A-B′), E9.5 (C-D″) and E10.5 (E-F′) does not indicate any increase in the number of apoptotic cells (indicated by black arrows, A-F′) in the neuroepithelium of *Pax3^Sp2H/Sp2H^* mutants (B,B′,D-D″,F,F′), compared with wild-type (*Pax3*^+/+^; A,A′,C-C″,E,E′) embryos at the stages encompassing neural tube closure (A,B, ventral view; A′,B′, dorsal view). The characteristic midline staining in the closed mid-forebrain of +/+ embryos (black arrowhead and arrows in C′ and E′, respectively) appears to be lacking in *Pax3^Sp2H/Sp2H^* embryos (D′,F′), although staining is present at the rostral limit of the forebrain (anterior neural ridge; black arrow in C′,D′), lateral midbrain (white asterisks, E,F) and lateral hindbrain (black arrows in C,D and C″,D″), as well as the developing somites (white arrow in E,F). Regions of failed cranial neural tube closure lie between the white arrowheads in D and F' and regions of failed spinal closure lie between the black arrowheads in F. Scale bars: 0.1 mm. SB, spina bifida. (G) Quantitative analysis of cleaved caspase-3 immunostaining in the cranial neuroepithelium reveals no difference between genotypes. (Black circles represent single embryos with mean±s.d. of the data shown in bars. Data for each embryo are from two to three sections; overall 15-37 sections per genotype.) See also Fig. S1.

In *Pax3^+/+^* and *Pax3^Sp2H/Sp2H^* embryos, TUNEL-positive cells were detected in the rostral forebrain, in the midline of the closed forebrain neural tube and in the hindbrain neural folds (Fig. 1A-F), corresponding to known sites of apoptosis in wild-type embryos (Massa et al., 2009; Mirkes et al., 2001). However, we did not observe an increase in the number or location of TUNEL-positive cells in the neural folds of *Pax3^Sp2H/Sp2H^* embryos at any stage of closure in either the cranial or spinal region (Fig. 1; Fig. S1). Consistent with the results of TUNEL staining, the number of cleaved caspase-3-positive, apoptotic cells in the cranial neural folds did not differ between genotypes (Fig. 1G).

**Fig. 2.**
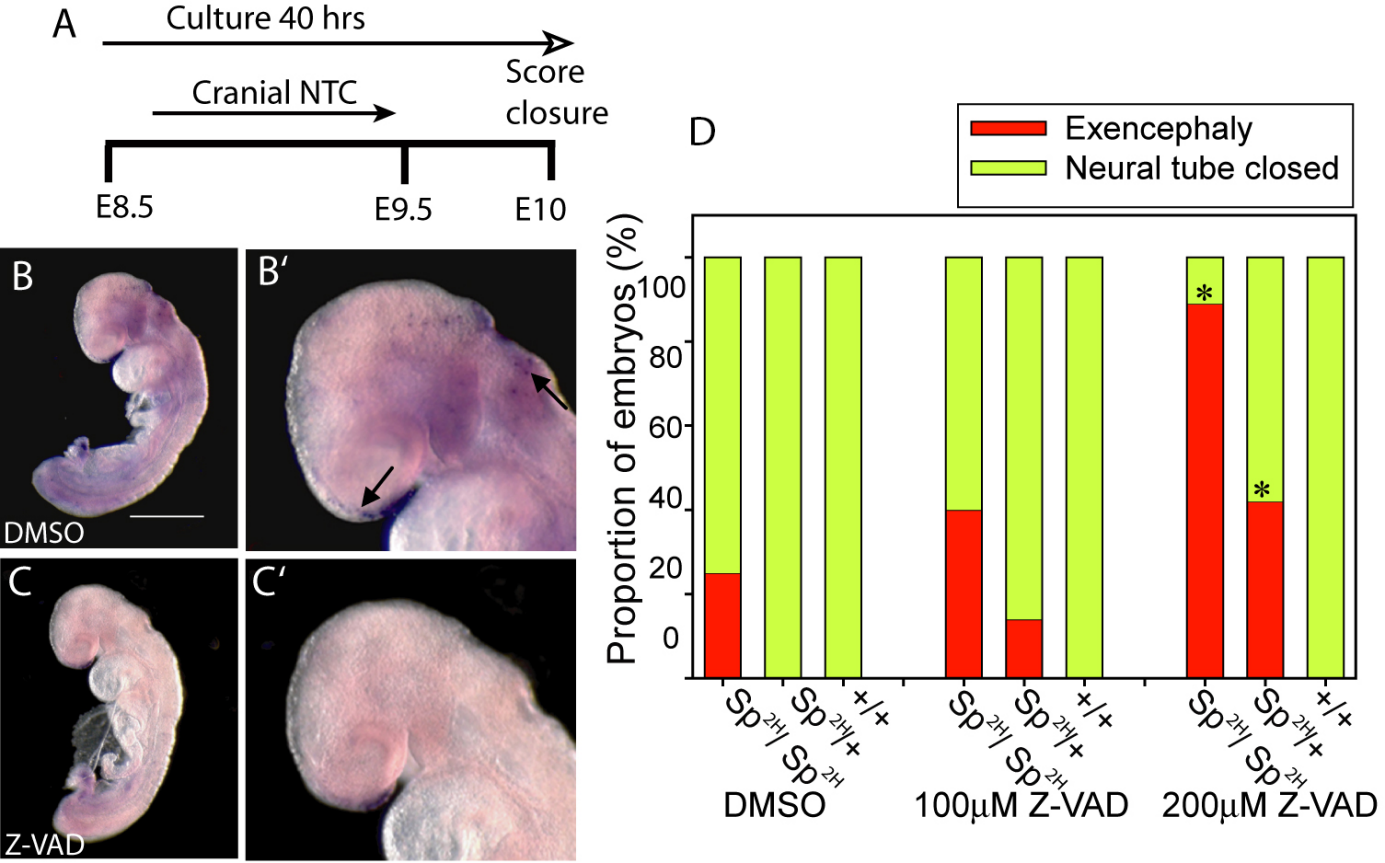
**NTDs in *Pax3* mutant embryos are not prevented by apoptosis inhibition.** (A-D) Treatment of cultured embryos with Z-VAD-FMK (strategy shown in A) did not impede cranial neural tube closure (NTC) in +/+ embryos (D), but led to increased frequency of exencephaly among *Pax3^Sp2H/^^Sp2H^* and *Pax3^Sp2H/^*^+^ embryos compared with vehicle (DMSO)-treated controls of the same genotype (*P<0.01; Fisher's exact test). TUNEL staining confirmed that apoptosis was suppressed in Z-VAD-treated embryos (C) compared with vehicle-treated controls (B) (apoptotic cells indicated by the arrow in B′). *n*=5-12 embryos per genotype for each treatment group (see Table S1 for numbers of embryos and measures of developmental progression and growth). Scale bar: 500 μm.

Although not supported by our TUNEL and cleaved caspase-3 analysis, we reasoned that if apoptosis is indeed causally related to *splotch* NTDs then direct inhibition of apoptosis would potentially rescue these defects. Therefore, as a complementary approach, embryos were cultured through the period encompassing cranial neurulation (40 h from E8.5) in the presence of the pan-caspase inhibitor Z-VAD-FMK, which we previously showed to effectively suppress apoptosis in neurulation-stage embryos (Massa et al., 2009) (Fig. 2). Contrary to predictions, inhibiting apoptosis caused a dose-dependent increase in exencephaly incidence among *Pax3^Sp2H/Sp2H^* embryos. These defects were also observed among some heterozygotes, which are unaffected in the absence of Z-VAD-FMK (Fig. 2D). In addition, the posterior neuropore (PNP) length in *Sp^2H/+^* embryos treated with 200 µM Z-VAD-FMK was greater than that among vehicle-treated controls, suggesting suppression of spinal neural tube closure (Table S1). Wild-type embryos were unaffected by the inhibitor. Moreover, there was no apparent effect of Z-VAD-FMK on viability, growth or developmental progression in *Sp^2H/+^* embryos treated with 200 µM Z-VAD-FMK compared with vehicle-treated controls, showing that increased exencephaly frequency does not result from generalised non-specific teratogenicity (Table S1). Hence, inhibition of apoptosis does not prevent NTDs and may even interact with genetic mutation of *Pax3* to increase the susceptibility to NTDs in embryos carrying the *Sp^2H^* allele. Overall, our data do not support the hypothesis that NTDs result from increased apoptosis in *Pax3* mutant embryos.

### *Pax3* mutation results in premature neuronal differentiation in the cranial neuroepithelium prior to failure of closure

**Fig. 3.**
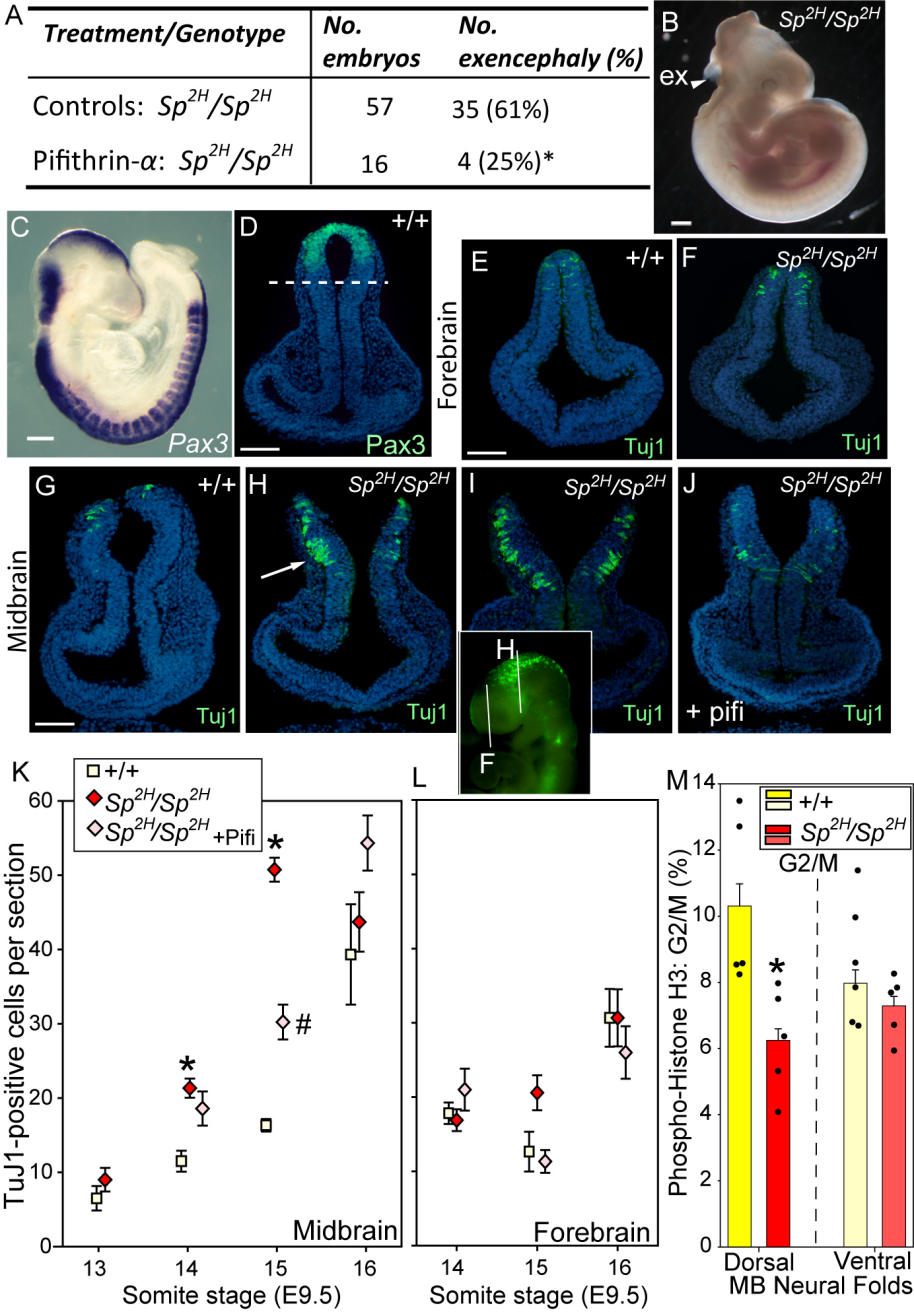
**Premature neuronal differentiation and diminished proliferation in *Pax3* mutant midbrain neuroepithelium.** (A,B) Among *Pax3^Sp2H/2H^* embryos exposed to pifithrin-α (via maternal administration), the frequency of exencephaly (arrowhead in B) was significantly lower than that among vehicle-treated controls (*P<0.02; Fisher's exact test). Data are from ten treated and 37 control litters; see Table S2 for additional details. (C,D) *Pax3* mRNA (C) and protein (D) are expressed in the dorsal neural tube (D, above dashed line on coronal section through the midbrain). (E-J) β-tubulin type III (Tuj1)-positive cells (as indicated by arrow in H) were analysed on coronal sections through the forebrain (E,F) and midbrain (G-J) at E9-E9.5 (positions of sections in H and K shown on inset whole-mount image). Scale bars: 500 µm in B,C; 100 µm in D,E,G. (G-I,K) In *Pax3^Sp2H/2H^* embryos, the midbrain neuroepithelium exhibits significantly more neuronal cells than that in wild type at the 14- to 15-somite stages (*P<0.002, compared with wild-type embryos at the same somite stage). (J,K) Pifithrin-α treatment results in a significantly reduced number of TuJ1-positive cells in the midbrain of *Pax3^Sp2H/2H^* embryos at the 15-somite stage (^#^*P*<0.002, compared with both +/+ and untreated *Sp^2H^/Sp^2H^* embryos). (L) No difference between genotype or treatment group were observed in the forebrain neuroepithelium. Each data point represents mean±s.e.m. for nine to 12 sections from a minimum of three embryos. (M) The proportion of PHH3-positive (late-G2- to M-phase) cells was significantly lower in the dorsal neuroepithelium of the midbrain of *Pax3^Sp2H/2H^* embryos compared with +/+ littermates at E9.5 (14- to 15-somite stage; *P<0.01, ANOVA). Black circles (*n*=5 embryos per genotype) represent single embryos with mean±s.e.m. of the data shown in bars (data for each embryo are from five sections, *n*=25 sections per genotype).

We next asked whether the frequency of cranial NTDs in *Pax3^Sp2H/Sp2H^* embryos can be lowered by maternal treatment with pifithrin-α, as described for embryos homozygous for the *Sp* allele of *Pax3* (Pani et al., 2002). We found a lower frequency of cranial NTDs (Fig. 3A,B) among *Pax3^Sp2H/Sp2H^* offspring of pifithrin-α treated mice compared with untreated control litters (Fig. 3A; Table S2).

Pifithrin-α was originally identified in a screen for inhibitors of p53 (Murphy et al., 2004), suggesting this as a mode of action in *Pax3* mutants. Surprisingly, however, among litters generated by intercross of *Pax3^Sp2H/+^; Trp53^+/−^* mice, we found no effect of p53 genotype on the susceptibility to NTDs in *Pax3^Sp2H/Sp2H^* embryos (Table 1). Cranial NTDs occurred among some *Trp53^−/−^* embryos that were wild type, as predicted from previous reports (Sah et al., 1995), demonstrating effectiveness of the *Trp53* mutant allele. Moreover, *Trp53* mutation caused an increased rate of cranial NTDs in *Pax3^Sp2H/+^* heterozygotes (Table 1). Lack of replication of the reported NTD prevention in *Pax3^Sp/Sp^; Trp53^−/−^* embryos (Pani et al., 2002) could potentially reflect an allele-specific difference between *Sp*, which primarily causes spinal NTDs, and *Sp^2H^*, which causes both cranial NTDs (∼60% of homozygotes) and almost fully penetrant spina bifida (Burren et al., 2008; Greene et al., 2009).

**Table 1.**
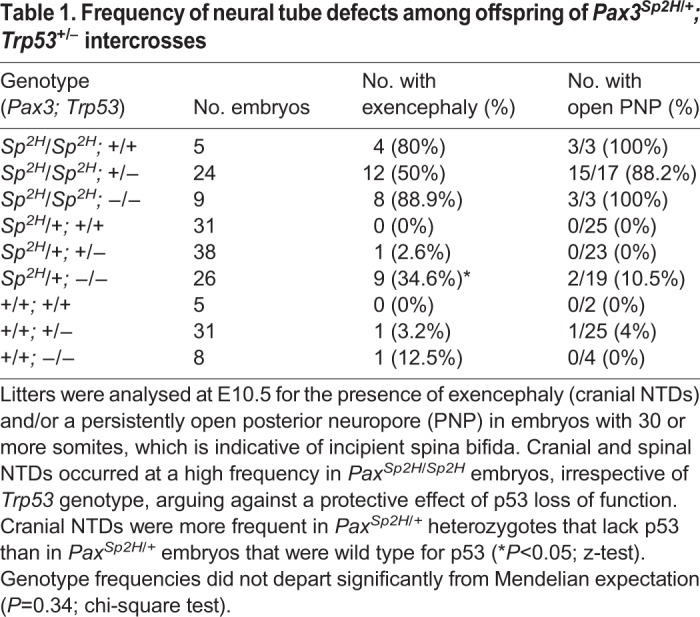
**Frequency of neural tube defects among offspring of *Pax3^Sp2H/+^; Trp53^+/−^*** **intercrosses**

Possible cellular responses to p53 activity include not only apoptosis but also cell-cycle arrest, senescence and differentiation (Vousden and Lane, 2007). Therefore, in view of the inconsistent findings with apoptosis inhibition, pifithrin-α usage and genetic knockout of p53, we asked whether cellular differentiation and/or cell-cycle progression may be dysregulated in the neuroepithelium of *Pax3* mutant embryos.

In wild-type embryos, the onset of neuronal differentiation correlated closely with the timing of cranial neural tube closure at E9.5 (Fig. 3). Few cells were positive for the early neuronal marker, β-tubulin type III (TuJ1; also known as Tubb3), in the midbrain neuroepithelium at the 13- to 15-somite stages, prior to closure (Fig. 3G,K). Neuronal differentiation then increased concomitantly with completion of cranial neural tube closure, which is achieved in wild-type embryos by the 16-somite stage (Fig. 3K). In contrast, *Pax3^Sp2H/Sp2H^* embryos showed a pronounced shift in the timing of neuronal differentiation in the midbrain, compared with wild-type embryos, with 83% and 210% increases in the number of Tuj1-positive cells at the 14- and 15-somite stages, respectively (Fig. 3H,I,K). These supernumerary neuronal cells arose in the dorsal region of neuroepithelium corresponding to the Pax3 expression domain (Fig. 3C,D; Fig. S2), suggesting that differentiation occurred as a result of loss of Pax3 function. Hence, excess neuronal cells were present in the midbrain neuroepithelium prior to failure of closure and at a stage when they are scarce in wild-type controls. Forebrain closure occurs successfully in *Pax3* mutant embryos, the forebrain being a region in which *Pax3* expression is weaker than at other axial levels of the neural tube in wild types (Fig. 3B,C). This region exhibited low numbers of Tuj1-positive cells at E9.5 with no difference between genotypes (Fig. 3E,F,L).

Among pifithrin-α treated embryos, concomitant with prevention of exencephaly in *Pax3^Sp2H/Sp2H^* embryos, there was a significant reduction in the number of TuJ1-positive cells in the midbrain neuroepithelium (Fig. 3J,K). Thus, our findings suggest that normalisation of neural tube closure in *Pax3* mutant embryos following pifithrin-α treatment may be mediated, not via prevention of p53-dependent apoptosis, but through suppression of premature neuronal differentiation.

### Impaired cell-cycle progression in *Pax3*-deficient neuroepithelium

We investigated the possibility that premature neuronal differentiation could be associated with altered proliferation in the neuroepithelium of *Pax3^Sp2H/Sp2H^* embryos. At neurulation stages, *Pax3^Sp2H/Sp2H^* embryos had a comparable number of somites and crown-rump length to those of wild-type littermates, showing that there is no overall growth or developmental retardation (Table S3). However, we observed a significant deficit in the proportion of phospho-histone H3 (PHH3)-labelled cells at late-G2/M phase in the dorsal neural plate of *Pax3^Sp2H/Sp2H^* embryos (Fig. 3M). Hence, a dorsal-high, ventral-low proliferation differential was observed in the midbrain neuroepithelium of wild-type embryos at E9.5, consistent with findings in the spinal neural tube (Kim et al., 2007; McShane et al., 2015; Megason and McMahon, 2002), whereas this proliferation differential was absent in *Pax3^Sp2H/Sp2H^* mutants (Fig. 3M).

### Failure of spinal neurulation is associated with diminished proliferation but not premature neuronal differentiation

**Fig. 4.**
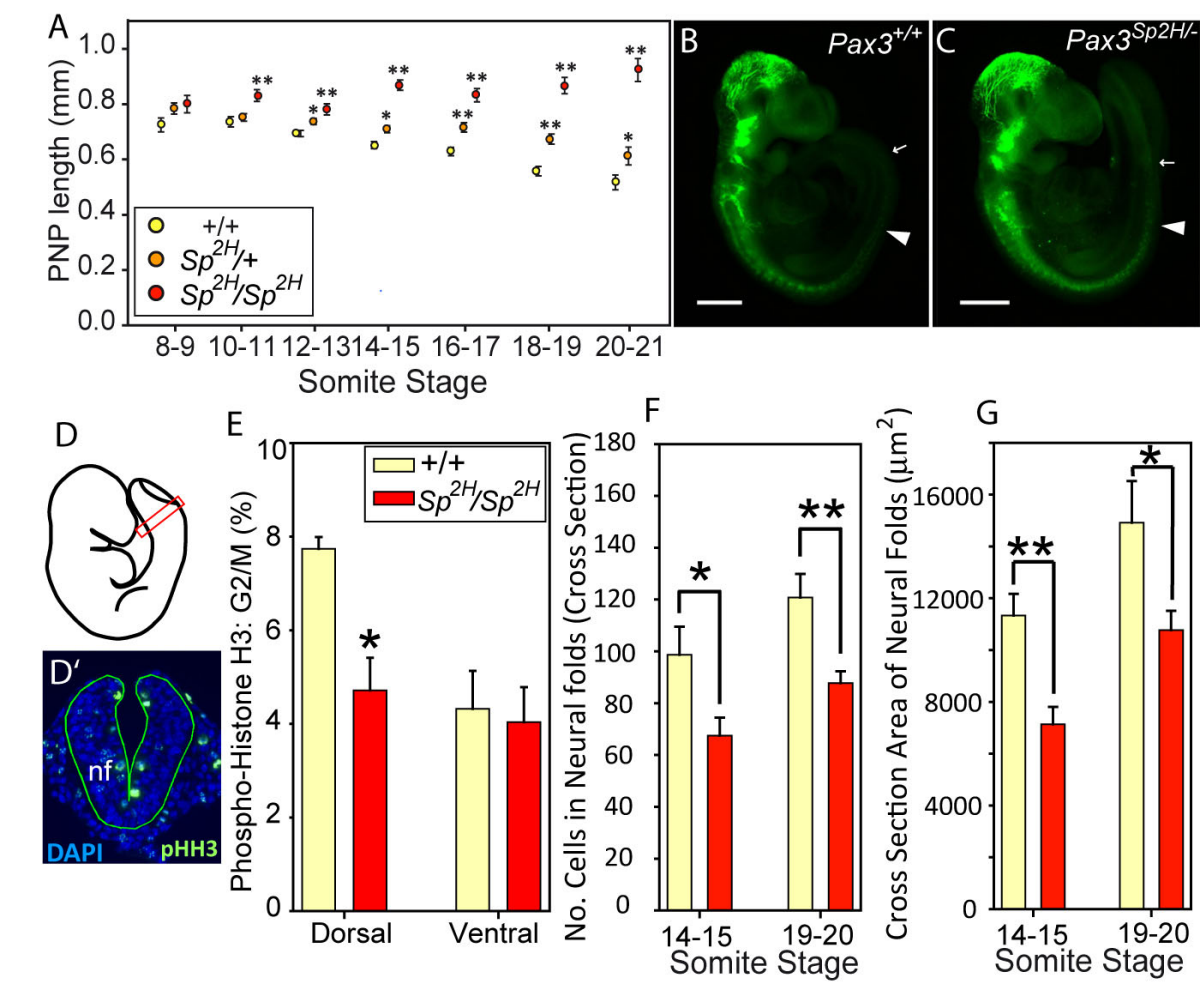
**Diminished proliferation and altered dimensions in the spinal neural tube of *Pax3^Sp2H/2H^* embryos.** (A) The PNP length of *Pax3^Sp2H/2H^* embryos is enlarged from E9 (10- to 11-somite stage) onwards. Delayed PNP closure is also evident in *Pax3^Sp2H/+^* embryos compared with stage-matched +/+ controls (*P<0.05, **P<0.01; ANOVA). (B,C) Staining for βIII-tubulin is detected in the spinal neural tube at E9.5 but the caudal limit (indicated by white arrowheads) does not approach the open PNP region (white arrows). See also Fig. S3. (D-G) Analysis of transverse sections (D′) at the level of the closing neural folds (nf; boxed area in D) at E9.5 (14- to 15-somite stage) shows a significantly lower proportion of PHH3-positive (late-G2 to M-phase) cells in the *Pax3* mutant dorsal neuroepithelium of *Pax3^Sp2H/2H^* compared with +/+ embryos (*P<0.01; Student's *t*-test) (E). The neuroepithelium of *Pax3^Sp2H/2H^* embryos displays a significantly lower number of cells (F) and cross-sectional area (G) than in +/+ embryos at both 14- to 15- and 19- to 20-somite stages (*P<0.05; **P<0.01). Bars in E-G represent mean±s.e.m. from six embryos per group (genotype and stage), with five to six sections per embryo (each bar corresponds to 32-36 sections). Scale bars: 500 μm.

The observations of premature neuronal differentiation and diminished proliferation in the dorsal cranial neuroepithelium at the stage of closure suggest potential causative mechanisms for cranial NTDs. We therefore asked whether comparable phenotypes were present in the spinal neural folds. Spina bifida occurs in almost all *Pax3^Sp2H/Sp2H^* embryos, and results from failure of PNP closure at E10.5. Measurements of PNP length showed that spinal neural tube closure was delayed from soon after initiation of closure, with an enlarged PNP evident at E9.0 (12- to 13-somite stage) and becoming particularly prominent from E9.5 (14- to 16-somite stage) onwards (Fig. 4A; Fig. S2E). The PNP did not dramatically increase in size as development progressed, unlike in mutants such as *Grhl3^−/−^* (de Castro et al., 2018), indicating that closure progresses in *Pax3* mutants. However, this is at a diminished speed such that closure ultimately fails to be completed in the low spine.

Arguing against a primary effect in which the presence of neuronal cells causes spina bifida, abnormal neuronal differentiation was not apparent in the spinal neuroepithelium of *Pax3* mutants at E9.5. Neuronal differentiation proceeds in a rostro-caudal temporal wave and, in embryos of all genotypes, Tuj1 staining (whole mount and on sections) was apparent only in the closed neural tube rostral to the site of PNP zippering. Neurons were never observed in the open PNP region of *Pax3^+/+^* (*n*=6), *Pax3^Sp2H/Sp2H^* (*n*=7) or *Pax3^Sp2H/+^* (*n*=7) embryos (Fig. 4B,C; Fig. S3). Moreover, the caudal axial level at which Tuj1-positive cells was detected in the closed neural tube did not differ between genotypes (Fig. S3).

As observed in the cranial region (Fig. 3M), the dorsal neuroepithelium of *Pax3^Sp2H/Sp2H^* embryos exhibited an apparent proliferation defect. Hence, at the 14- to 15-somite stage (E9.5), soon after delay of PNP closure became apparent, immunostaining for PHH3 at the rostral level of the PNP revealed a significant decrease in the proportion of cells at late-G2/M phase in the dorsal neural plate (Fig. 4D-E), corresponding to the region of *Pax3* loss of function (Fig. S2B). This proliferation deficit abolished the dorsal-ventral proliferation difference that is present in wild-type embryos at this stage (Fig. 4E; Fig. S4A,B) and led to a significant reduction in the number of cells in the neuroepithelium (Fig. 4F), including in both dorsal and ventral subregions (Fig. S4C-E). In parallel with this finding, the cross-sectional area of the neuroepithelium was diminished in *Pax3* mutants compared with wild-type embryos (Fig. 4G), as were the dorsoventral height and mediolateral width of the neural tube, but not their relative ratio (Fig. S4G-J). The significant deficit of cells and size of the *Pax3* mutant neural plate, detected in 14- to 15-somite-stage embryos, was still evident at the 19- to 20-somite stage (Fig. 4F,G; Fig. S4). These findings show that suppression of PNP closure is associated with a proliferation defect in the dorsal neuroepithelium.

Overall, *Pax3* loss of function in the spinal region causes diminished proliferation without induction of neuronal differentiation. It seems likely that Pax3 has comparable functions at cranial and spinal levels. Hence, the effect of Pax3 on the balance between proliferation and differentiation in the cranial region, where the neuroepithelium is primed to differentiate, may be principally mediated through regulation of cell-cycle progression rather than direct inhibition of differentiation.

### In *Pax3* mutants, FA acts to increase cell proliferation by promoting S-phase to G2 progression

**Fig. 5.**
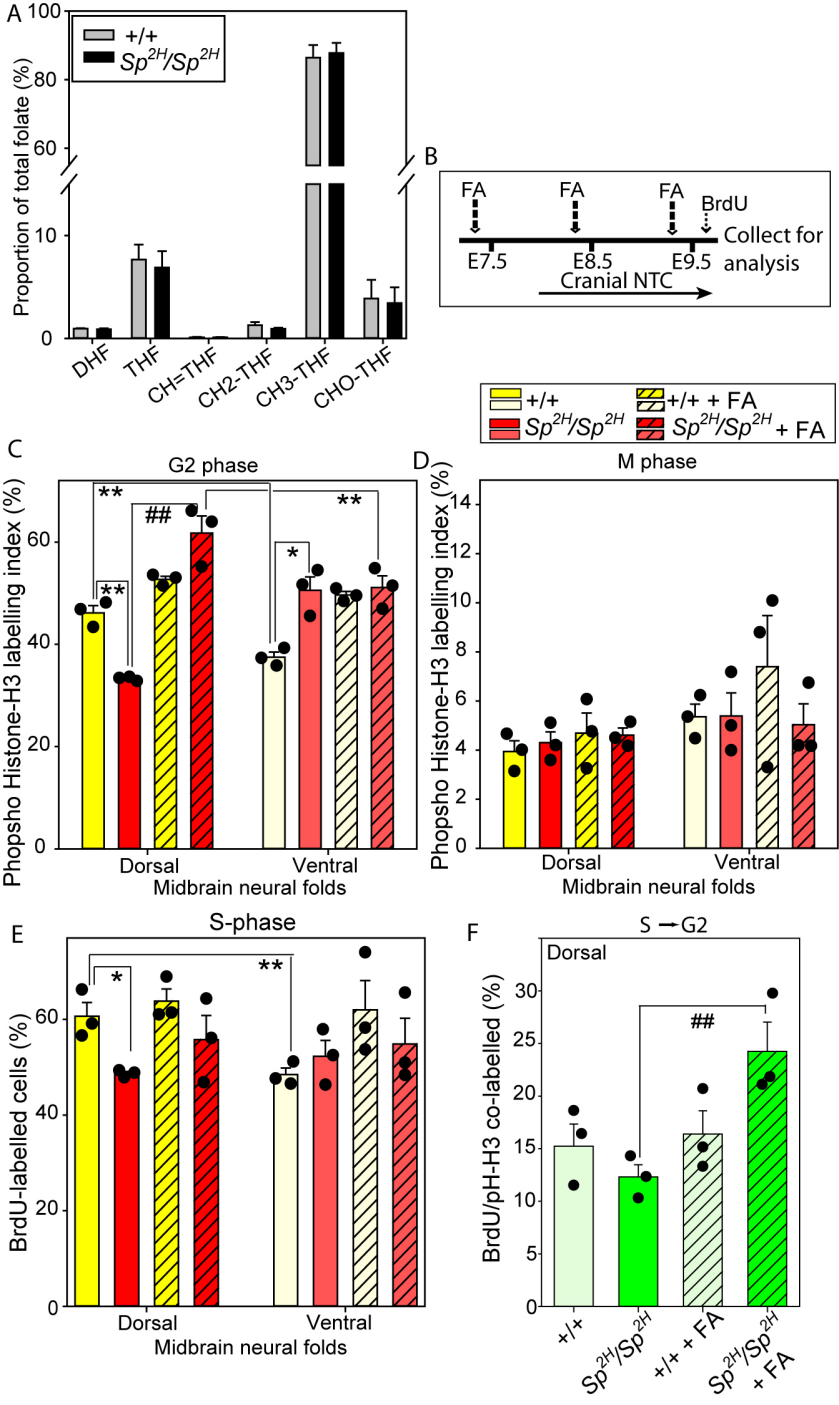
**FA promotes cell-cycle progression in the *Pax3* mutant neuroepithelium.** (A) The relative proportion of differing folate species, separated by mass spectrometry, did not differ with genotype (DHF, dihydrofolate; THF, tetrahydrofolate; CH=THF, methenyl-THF; CH2, methylene; CH3, methyl; CHO, formyl-THF) (*n*=4 embryos per genotype). (B) Scheme for *in vivo* treatment with FA prior to embryo collection with analysis of the midbrain neuroepithelium for PHH3-labelled cells in (C) G2 phase and (D) M phase, or (E) BrdU labelling of S phase. NTC, neural tube closure. (F) Proportion of BrdU/PHH3 co-labelled cells in the dorsal neuroepithelium. Significant differences between genotypes, or between dorsal and ventral regions within treatment groups, are indicated (*P<0.025; **P<0.0025; ANOVA). FA treatment led to increased PHH3 (C) and PHH3/BrdU double labelling (F) in the dorsal neuroepithelium of *Pax3* mutants (^##^*P*<0.01; ANOVA).

*splotch* (*Pax3^Sp2H^*) was the first mouse model in which NTDs were found to be preventable by supplemental FA (Fleming and Copp, 1998), but – as in human NTDs – the protective mechanism has not been defined. FA is reduced via dihydrofolate to THF, which acts to carry 1C groups in folate 1C metabolism (FOCM), a key function of which is to provide 1C units for nucleotide biosynthesis. Our previous analysis showed no effect of *Pax3^Sp2H^* genotype on embryonic folate content at neurulation stages (Burren et al., 2008). Here, mass-spectrometry-based analysis of the individual folate species that are involved in FOCM did not reveal significant perturbation in their relative abundance in *Pax3^Sp2H/Sp2H^* embryos compared with wild type (Fig. 5A). This is consistent with our finding that the relative abundance of s-adenosylhomocysteine and s-adenosylmethionine did not differ with *Pax3^Sp2H^* genotype (Burren et al., 2008). Moreover, unlike loss-of-function mutants of the mitochondrial FOCM components Mthfd1L and Gldc, or the mitochondrial folate transporter SLC25A32 (Momb et al., 2013; Pai et al., 2015; Leung et al., 2017; Kim et al., 2018), NTDs were not prevented by maternal supplementation with formate, a 1C donor, in *Pax3* mutants (Table S4).

Cranial neurulation appears highly sensitive to disturbance of cellular proliferation, with exencephaly often being induced by teratogens that cause generalised retardation of embryonic growth or developmental progression (Copp, 2005). Together with our finding of a proliferation defect in the Pax3-deficient cranial and spinal neuroepithelium, this prompted the question of whether FA status impacts cell proliferation in developing *Pax3^Sp2H/Sp2H^* embryos. Indeed, FA is required for proliferation of cultured neural progenitor cells (Ichi et al., 2010, 2012). We therefore carried out further analysis of cell-cycle progression in the *Pax3* mutant neural plate in order to determine whether maternal FA treatment could influence cell-cycle progression. Dams were treated with FA using the protocol that we found to reduce the frequency of cranial NTDs among *Pax3^Sp2H/Sp2H^* offspring (Burren et al., 2008; Fleming and Copp, 1998). Embryos were analysed during the period of cranial neurulation at E9.5 (14- to 15-somite stage), with a pulse of bromodeoxyuridine (BrdU) preceding embryo collection to allow analysis of cell-cycle progression in the midbrain neuroepithelium (Fig. 5B).

The proportion of cells in G2 and M phase of the cell cycle were analysed on the basis of the characteristic appearance of PHH3 immunostaining (de Castro et al., 2012). A lower proportion of non-mitotic PHH3-positive (early-G2 to late-G2) cells was present in the dorsal neuroepithelium of *Pax3^Sp2H/Sp2H^* embryos compared with +/+ littermates (Fig. 5C), corresponding with the observed deficit of cells at late-G2/M phase (Fig. 3M). In contrast, the mitotic (M-phase) population did not differ between genotypes (Fig. 5D). Interestingly, there was a small but significantly higher proportion of G2-phase cells in the ventral neuroepithelium of *Pax3* mutant compared with +/+ embryos, which suggests a possible non-cell-autonomous effect of Pax3 loss of function.

The BrdU-labelling index (reporting cells in S phase) was also significantly diminished in the dorsal neuroepithelium of *Pax3^Sp2H/Sp2H^* embryos (Fig. 5E), whereas the ventral region did not differ from wild type (Fig. 5E). Hence, both BrdU labelling and PHH3 immunostaining showed the presence of a dorsal-high to ventral-low proliferation differential in wild-type embryos that is absent, or even reversed, in *Pax3* mutants (Fig. 5D,E).

FA treatment had a striking effect on cell-cycle parameters, particularly in the dorsal midbrain neuroepithelium of *Pax3^Sp2H/Sp2H^* embryos, where an FA-induced increase in the proportion of PHH3-positive, G2-phase cells was observed (Fig. 5C). Similarly, although FA did not significantly alter the proportion of S-phase, BrdU-labelled cells, there was a trend towards increased labelling in all regions (Fig. 5E). Most notably, in FA-treated *Pax3^Sp2H/Sp2H^* embryos, we observed a significant increase in the proportion of double-positive BrdU/PHH3 cells; i.e. cells that were in S phase during the pulse of BrdU labelling and then progressed into G2 by the time of embryo collection (Fig. 5F). Hence, the rate of transition from S phase to G2 was increased by supplemental FA.

In the dorsal and ventral neuroepithelium of *Pax3^+/+^* embryos, FA treatment led to non-significant trends towards increased labelling for both PHH3 (Fig. 5C) and BrdU (Fig. 5E). As a result, the dorsal-ventral proliferation differential was absent in FA-treated +/+ embryos. In contrast, the proportion of cells in M phase did not differ with FA treatment in either *Pax3* genotype (Fig. 5D).

## DISCUSSION

The *Pax3* mutant (*Sp^2H^*) mouse provides a model in which to investigate the cause of NTDs that are sensitive to folate deficiency and responsive to prevention by FA. Analysis of apoptosis markers, together with lack of NTD prevention by inhibition of apoptosis, are incompatible with the idea that increased cell death contributes to failure of neural tube closure. Instead, our findings suggest that Pax3 is required for cell-cycle progression and suppression of neuronal differentiation in the dorsal neuroepithelium until completion of cranial neural tube closure. The close correlation between onset of neuronal differentiation and completion of cranial neural tube closure that is observed in wild-type embryos is lost in *Pax3* mutants. Consistent with these observations, anti-sense downregulation of *Pax3* in cultured neuronal ND7 cells resulted in morphological differentiation, without apparent cell death (Reeves et al., 1999). Similarly, during melanogenesis, Pax3 induces the melanocyte fate but acts to repress terminal differentiation until additional differentiation signals are present (Lang et al., 2005). Moreover, during myogenesis, *Pax3* is required in muscle progenitors but must be downregulated for myoblast differentiation (Goljanek-Whysall et al., 2011). Thus, in different cellular contexts, including the neuroepithelium, Pax3 may function to maintain committed progenitor cells in an undifferentiated, proliferative state until an appropriate developmental stage.

The relationship between *Pax3* mutation, cell-cycle regulation and folate status is intriguing. Function of FOCM depends on adequate abundance of the THF ‘backbone’ (from maternal diet and/or microbiota) and supply of 1C units. Supplemental FA can enhance supply of THF but does not carry a 1C unit. During neurulation, the primary requirement for 1C units is met by catabolism of serine and glycine in mitochondrial FOCM (Leung et al., 2017; Tibbetts and Appling, 2010). This generates formate, which is transferred to cytoplasmic FOCM, in which the intermediates 5,10-methylene THF and 10-formyl THF act as the 1C donors in thymidylate and purine biosynthesis, respectively. Conditions of folate deficiency further impose a requirement for serine-dependent generation of 5,10-methylene THF by the action of SHMT1 (Beaudin et al., 2011), acting in the cytoplasm and/or nucleus (Herbig et al., 2002; MacFarlane et al., 2011).

*Pax3* genotype does not affect embryonic folate content at neurulation stages (Burren et al., 2008) or the relative proportion of different folate intermediates (the current study). This contrasts with mouse embryos carrying genetic defects in FOCM-associated enzymes such as *Mthfr* (Leung et al., 2017) and *Gldc* (Pai et al., 2015), or with methotrexate exposure (a FOCM inhibitor) (Leung et al., 2013). Unlike in *Pax3* mutants, NTDs resulting from disruption of the 1C supply from mitochondrial FOCM (e.g. by mutation of Gldc or Amt) are neither FA preventable nor responsive to folate deficiency (Narisawa et al., 2012; Pai et al., 2015). Furthermore, although NTDs in *Gldc*- and *Mthfd1L*-null embryos are preventable by maternal supplementation with formate as a 1C donor (Leung et al., 2017; Momb et al., 2013; Pai et al., 2015), we found no apparent protective effect of this treatment in *Pax3* mutants. These findings highlight the potential for non-equivalent mechanisms underlying FA-mediated prevention of NTDs compared with prevention by 1C donors (e.g. formate) of NTDs resulting from disruption of FOCM.

Overall, it appears unlikely that NTDs in *Pax3* (*splotch*) embryos result from a deficit in folate uptake, 1C supply or interconversion of folate intermediates in FOCM. Significantly, however, *Pax3* mutant embryos and embryonic fibroblasts preferentially use the nucleotide salvage pathway over *de novo* thymidylate biosynthesis, suggesting a possible impairment of FOCM that could underlie sensitivity to maternal folate status (Beaudin et al., 2011; Burren et al., 2008; Fleming and Copp, 1998). Moreover, the mutual NTD-exacerbating pairwise interactions of folate deficiency, *Shmt1* loss of function and *Pax3* mutation further implicate thymidylate synthase as an FA-sensitive process for neural tube closure (Beaudin et al., 2011; Burren et al., 2008; Martiniova et al., 2015). For example, folate deficiency or *Pax3* mutation can cause NTDs in *Shmt1*-null embryos, which do not exhibit NTDs on a folate-replete diet (Beaudin et al., 2011). The apparent deficit of *de novo* thymidylate synthesis in *Pax3^Sp2H^* mutants, under normal dietary conditions, is not due to a limiting pool of 5,10-methylene THF, but may instead relate to post-transcriptional downregulation of thymidylate synthase, as found in *Pax3^Sp^* mutant embryos (Beaudin et al., 2011). A lowering of THF availability in folate-deficient conditions could then further suppress thymidylate synthesis and exacerbate NTDs. This would also be consistent with the increased frequency of *Pax3*-related NTDs caused by loss of function of SHMT1, the translocation of which to the nucleus favours use of 5,10-methylene THF in thymidylate synthesis over reduction to 5-methyl THF for methylation of homocysteine (Herbig et al., 2002).

We previously found that maternal dietary folate deficiency limits embryonic growth and developmental progression of wild-type and *Pax3* mutant embryos without dissociating these parameters (Burren et al., 2008). Here, we find that supplemental FA stimulates cell-cycle progression, and that this effect is mediated via an increased rate of transition through S phase and into G2 phase. An effect of FA in the DNA-synthesis phase of the cell cycle correlates with a proposed mechanism of FA action in stimulating thymidylate biosynthesis.

Notably, the effect of FA in driving cell-cycle progression was greatest in *Pax3* mutant regions, such that the overall effect was to normalise the dorsal-ventral proliferation gradient in *Pax3^Sp2H/Sp2H^* embryos. It appears that dorsal proliferation, as opposed to the dorsal-ventral gradient specifically, is associated with NTD prevention, as FA-treated wild-type embryos no longer exhibit a proliferation gradient, but all complete closure. The particular requirement for cellular folate in the dorsal neuroepithelium is highlighted by the notable dorsal (overlapping the Pax3 expression domain) versus ventral enrichment of mRNA for the folate receptor, *Folr1* (Saitsu et al., 2003), the knockout of which also causes NTDs (Piedrahita et al., 1999). Overall, we hypothesise that supplemental FA contributes to neural tube closure by promoting S-phase cell-cycle progression, particularly in the neuroepithelial component of the dorsal neural folds.

## MATERIALS AND METHODS

### Mice and collection of embryos

*Pax^Sp2H^* (*Sp^2H^*; *splotch*) mice were maintained as a closed, random-bred (heterozygous with wild type) colony. The *Sp^2H^* mutation is carried on a mixed background, which includes CBA/Ca, 101 and C3H/He. Experimental litters were generated by intercross of heterozygous mice. Embryos were genotyped by PCR of genomic DNA (Conway et al., 1997). To generate mice lacking p53, *Trp53^flox/+^* mice (Jonkers et al., 2001) were used to generate *Trp53^+/−^* heterozygotes for intercross to *Pax3^Sp2H^*. The *Pax3^cre^* allele was used as a null allele in crosses with the p53-null allele (Engleka et al., 2005). Animal studies were carried out under regulations of the Animals (Scientific Procedures) Act 1986 of the UK Government, and in accordance with the guidance issued by the Medical Research Council, UK in *Responsibility in the Use of Animals for Medical Research* (July 1993).

Pifithrin-α (Calbiochem) treatment at a dose of 2.2 mg/kg was achieved by intraperitoneal injection with 0.22 mg/ml solution [diluted with phosphate buffered saline (PBS) from a 5 mg/ml stock in sterile dimethyl sulfoxide (DMSO)], at E8.5 and E9.5 (Pani et al., 2002). FA (20 mg/kg) was administered by intraperitoneal injection of dams at E7.5, E8.5 and E9.5, using a dose that we have found to lower the frequency of NTDs (Burren et al., 2008; Fleming and Copp, 1998). Embryos were collected 3 h after the final injection. Formate treatment was administered by addition of sodium formate (30 mg/ml) in the drinking water of the dam from E0.5 until the collection of litters (Pai et al., 2015). Litters were dissected from the uterus in Dulbecco's modified Eagle medium (DMEM) containing 10% fetal calf serum. Embryos were rinsed in PBS and fixed in 4% paraformaldehyde (PFA). Embryos were dehydrated in a methanol series and stored at −20°C prior to *in situ* hybridisation or TUNEL analysis.

For immunohistochemistry, BrdU (Invitrogen) was administered by maternal intraperitoneal injection at 50 mg/kg from a stock solution of 10 mg/ml. Litters were collected after 15 min, embryos explanted in ice-cold DMEM, immediately fixed in 4% PFA, dehydrated through an ethanol series and processed for wax embedding.

### Whole-embryo culture

Embryos were explanted at E8.5, leaving the yolk sac and ectoplacental cone intact and cultured for 40 h in rat serum at 38°C (Dunlevy et al., 2006; Pryor et al., 2012). Stock solutions of 50 mM and 100 mM Z-VAD-FMK (Sigma-Aldrich) in DMSO were added to cultures as 0.2% (v/v) additions. An equivalent volume of vehicle was added to control cultures, and embryos were randomly allocated to treatment groups.

### TUNEL analysis

TUNEL analysis of whole embryos (Dunlevy et al., 2006) was performed on a minimum of eight embryos of each genotype at E8.5, E9.0, E9.5 and E10.5.

### Whole-mount *in situ* hybridisation

Whole-mount *in situ* hybridisation (de Castro et al., 2018) was performed using sense and anti-sense digoxygenin-labelled riboprobes for *Pax3* (Conway et al., 1997), generated using a digoxygenin RNA-labelling kit (Roche) and purified on Chroma spin columns (Clontech).

### Immunohistochemistry

Immunostaining using antibodies for PHH3 (06-570 Merck Millipore) and activated caspase-3 (Cell Signaling Technology) was performed on paraffin-embedded coronal (cranial) or transverse (spinal) sections. Numbers of positive cells were counted in defined areas, blind to genotype (Dunlevy et al., 2006). The number of positive cells per section was normalised to the area of neuroepithelium (cells/1000 µm^2^) or the total number of neuroepithelial cells on the section as detected by 4′,6-diamidino-2-phenylindole (DAPI) staining (% labelling).

Double staining for PHH3 and BrdU was performed following the deparaffinisation of slides by HistoClear. Sections were rehydrated through an ethanol series and antigen retrieval was performed in 0.01 M citric acid (pH 6.0). After washing in PBS, 0.1% Triton X-100, slides were blocked in 5% sheep serum, 0.15% glycine, 2% bovine serum albumin (BSA), followed by addition of anti-PHH3 (1:300). Secondary antibody was Alexa Fluor 568 (A11077, Life Technologies), diluted 1:500 in blocking solution. Nuclear stain was DAPI, diluted in PBS (1:10,000). After post-fixation in 4% PFA, slides were incubated in 1 M HCl, followed by 2 M HCl, to expose the BrdU antigen, and neutralised by washing in 0.1 M sodium borate (pH 8.5). Slides were blocked in 10% sheep serum and incubated with anti-BrdU (347583, BD Bioscience; 1:100). After incubation with secondary antibody (Alexa Fluor 488, A11070, Life Technologies; 1:500), slides were mounted [12.5% Mowiol 4-88 (Sigma-Aldrich), 0.2 M Tris base, 0.2 M Tris-HCl, 30% glycerol, pH 6.8]. Images were acquired on an inverted LSM710 confocal microscope (Zeiss).

For TuJ1 (β-tubulin type III) staining, 15 µm coronal cryosections were prepared (Burns and Douarin, 1998). Sections were blocked (PBS, 10% heat-inactivated sheep serum, 0.1% Triton X-100) and treated with primary antibodies against TuJ1 (BAbCO; 1:1000 in blocking solution) or Pax3 (American Type Culture Collection; 1:50), followed by secondary antibody (goat anti-mouse Alexa Fluor 488, Southern Biotech; 1:500). Sections were mounted in Vectashield with DAPI (Vector Laboratories). The number of TuJ1-positive cells in three adjacent matched sections of forebrain and midbrain was counted for each embryo.

For whole-mount immunostaining, embryos were fixed in methanol/DMSO (4:1) and incubated in methanol/DMSO/30% hydrogen peroxide (4:1:1). Embryos were blocked in PBS containing 2% non-fat dried milk, 0.5% Triton X-100 and 10% sheep serum, and then incubated in the same solution with antibody against TuJ1 (1:1000) followed by secondary antibody (donkey anti-mouse Alexa Fluor 488, Invitrogen; 1:500).

### Analysis of folates by mass spectrometry

Analysis of multiple folates was performed by ultra-pressure liquid chromatography–tandem mass spectrometry as described previously (Leung et al., 2017; Pai et al., 2015). Folates were measured by multiple-reaction monitoring with optimised cone voltage and collision energy for precursor and product ions as described (Cabreiro et al., 2013; Leung et al., 2013).

Statistical analysis was performed using Sigmastat version 3.5 (Systat Software).

## Supplementary Material

Supplementary information
