# Supplementary material for: Cellular mechanisms underlying Pax3-related neural tube defects and their prevention by folic acid
Source: Dis Model Mech. 2019 Nov 22;12(11):dmm042234. doi: 10.1242/dmm.042234 (PMC6899032; doi:10.1242/dmm.042234)
Supplement: Supplementary information [file dmm-12-042234-s1.pdf]

Supplementary Material

Treatment/ Genotype	***No.***	***Exen***	***PNP length (mm)***	***Somites***	***Crown-rump length (mm)***	***Yolk sac circulation***
**Control-DMSO**						
Sp^{2H}/Sp^{2H}	12	3 (25%)	1.21 ±0.16*	24.9 ±0.3	3.29 ±0.09	2.6 ±0.1
$Sp^{2H}/+$	15	0	0.46 ±0.06	25.3 ±0.3	3.36 ±0.07	2.4 ±0.2
+/+	8	0	0.36 ±0.06	25.3 ±0.5	3.40 ±0.11	2.6 ±0.2
**100 µM ZVAD**						
Sp^{2H}/Sp^{2H}	5	2 (40%)	0.90 ±0.13*	25.0 ±0.4	3.25 ±0.12	2.8 ±0.2
$Sp^{2H}/+$	7	1 (14%)	0.53 ±0.09	25.1 ±0.6	3.25 ±0.12	2.9 ±0.1
+/+	8	0	0.28 ±0.04	25.9 ±0.6	3.27 ±0.13	2.3 ±0.3
**200 µM ZVAD**						
Sp^{2H}/Sp^{2H}	9	8 (89%)[#]	0.97 ±0.13*	24.3 ±0.6	3.11 ±0.12	2.0 ±0.3
$Sp^{2H}/+$	12	5 (42%)[#]	0.55 ±0.05**	24.4 ±0.7	3.17 ±0.12	2.0 ±0.3
+/+	12	0	0.33 ±0.03	24.5 ±0.5	3.12 ±0.10	2.0 ±0.2

Table S1. NTD incidence, growth and development of *splotch* embryos cultured in the presence of ZVAD-FMK or DMSO only (vehicle control). Embryos were cultured for 40 hours from E8.5-10. No significant differences were detected in yolk-sac circulation score (ranked from 0: no circulation to 3: vigorous circulation throughout the yolk sac), crown-rump length or number of somites after culture, suggesting that there was no adverse effect on viability, growth or developmental progression, respectively.

# Frequency of exencephaly (Exen) was signifcantly higher among embryos exposed to 200 µM ZVAD than among embryos of the same genotype exposed to DMSO ($p < 0.025$; Z-test).

* Mean posterior neuropore (PNP) lengths of Sp^{2H}/Sp^{2H} embryos were significantly larger than for heterozygous and wild-type embryos, reflecting the incipient spina bifida. ** The PNP was longer in $Sp^{2H}/+$ than in +/+ embryos and this was statistically significant in the 200 µM treatment group. No difference in PNP length was noted between treatment groups. Values are presented as mean ± SEM.

Treatment/Genotype	***No. litters***	***No. embryos***	***No. exencephaly (%)***	***No. spina bifida (%)***
**Controls**	37	225		
*Sp^2H^/Sp^2H^*		57	35 (61%)	20 (87%)
Sp^2H^/+		106	0 (0%)	0 (0%)
+/+		62	1 (2%)	0 (0%)
**Pifithrin-α**	10	60		
*Sp^2H^/Sp^2H^*		16	4 (25%)*	14 (88%)
Sp^2H^/+		27	0 (0%)	0 (0%)
+/+		17	0 (0%)	0 (0%)

Table S2. Incidence of neural tube defects among litters of *Splotch* (*Sp^2H^*) mice treated with pifithrin-α. Dams were treated with pifithrin-α at E8.5 and E9.5, by intra-peritoneal injection at a dose of 0.22 mg/kg (10 µl/g of a 2.2 mg/ml stock solution), and controls were injected with the same volume of PBS. Litters were collected at E11.5 and analysed for the presence of neural tube defects. Mean litter size did not significantly differ between treatment groups.* Frequency of exencephaly is significantly lower among *Sp^2H^/Sp^2H^* embryos exposed to pifithrin-α than among PBS-treated ($p<0.05$; Z-test with Yates correction), whereas spina bifida frequency is not affected by pifithrin-α.

Disease Models & Mechanisms • Supplementary information

Developmental Stage	***No. litters***	***Genotype***	***No. embryos***	***Somite number***	***Crown-rump length (mm)***
**E9.5**	14	+/+	22	15.7 ± 0.5	2.2 ± 0.2
		Sp^{2H}/+	43	15.1 ± 0.2	2.1 ± 0.1
		Sp^{2H}/Sp^{2H}	23	15.9 ± 0.3	2.2 ± 0.1
**E10.5**	6	+/+	11	30.9 ± 0.8	4.3 ± 0.1
		Sp^{2H}/+	17	30.0 ± 0.5	4.1 ± 0.1
		Sp^{2H}/Sp^{2H}	8	30.3 ± 0.6	4.2 ± 0.1

Table S3. Number of somites and crown-rump length do not differ between *splotch* (Sp^{2H}) genotypes. Litters were collected at E9.5 and E10.5, the number of somites was counted and the crown-rump length measured using an eye-piece graticule. No significant differences between genotypes were detected at either stage of development. Values are presented as mean ± SEM.

Disease Models & Mechanisms • Supplementary information

Genotype/treatment	***No. embryos***	***No. exencephaly (%)***	***No. spina bifida (%)***
**Formate**	33		
Sp^{2H}/Sp^{2H}	5	3 (60.0%)	5 (100%)
Sp^{2H}/+	18	0 (0%)	0 (0%)
+/+	10	0 (0%)	0 (0%)
**Controls**	168		
Sp^{2H}/Sp^{2H}	37	25 (67.6)	35 (94.6%)
Sp^{2H}/+	70	0 (0%)	0 (0%)
+/+	31	0 (0%)	0 (0%)

Table S4. Frequency of neural tube defects among offspring of formate-treated mice.

Litters were generated by intercross of *$Sp^{2H/+}$* mice. Dams were treated with formate (30 mg/ml in drinking water) and litters were analysed at E11.5 for the presence of exencephaly (cranial NTDs) and/or spina bifida.

Supplementary Figures

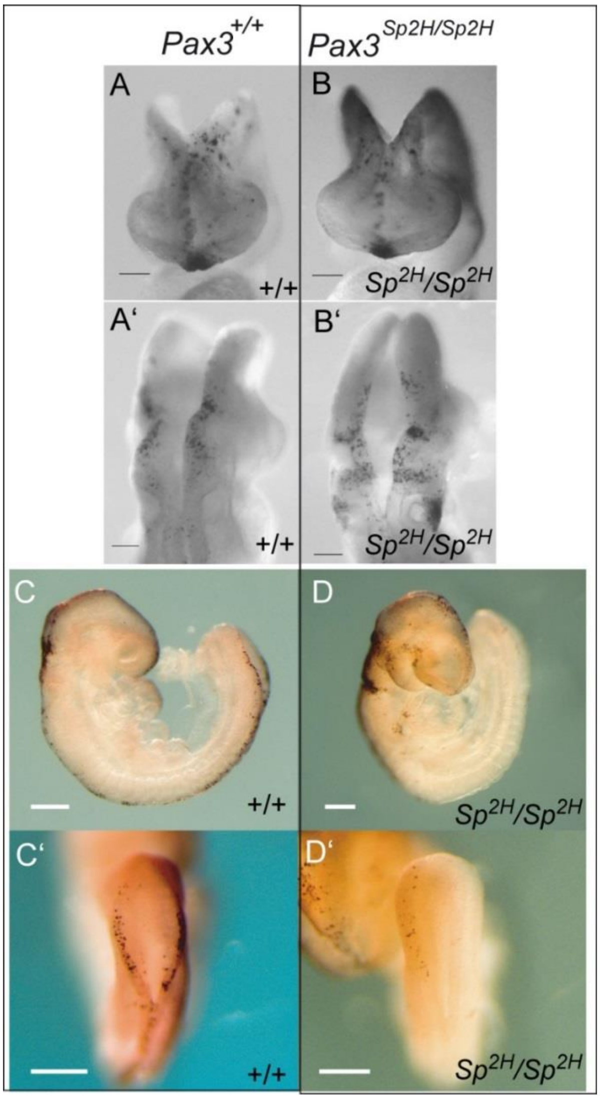

Figure S1. TUNEL staining of wild-type and *Pax3*$^{Sp2H/Sp2H}$ mutant embryos at E9.0. (A-B) No difference in numbers of TUNEL-positive apoptotic cells were detected in the neural folds of the cranial region (A, anterior view of forebrain; B, view of hindbrain). (C-D) There was no indication of excess TUNEL staining in the spinal region of *Pax3*$^{Sp2H/Sp2H}$ embryos, whereas some mutants exhibited fewer positive cells in the region of open spinal neural folds (compare D' and C'). Scale bar represents 0.1 mm (A-B), 0.25 mm (C-D).

Disease Models & Mechanisms • Supplementary information

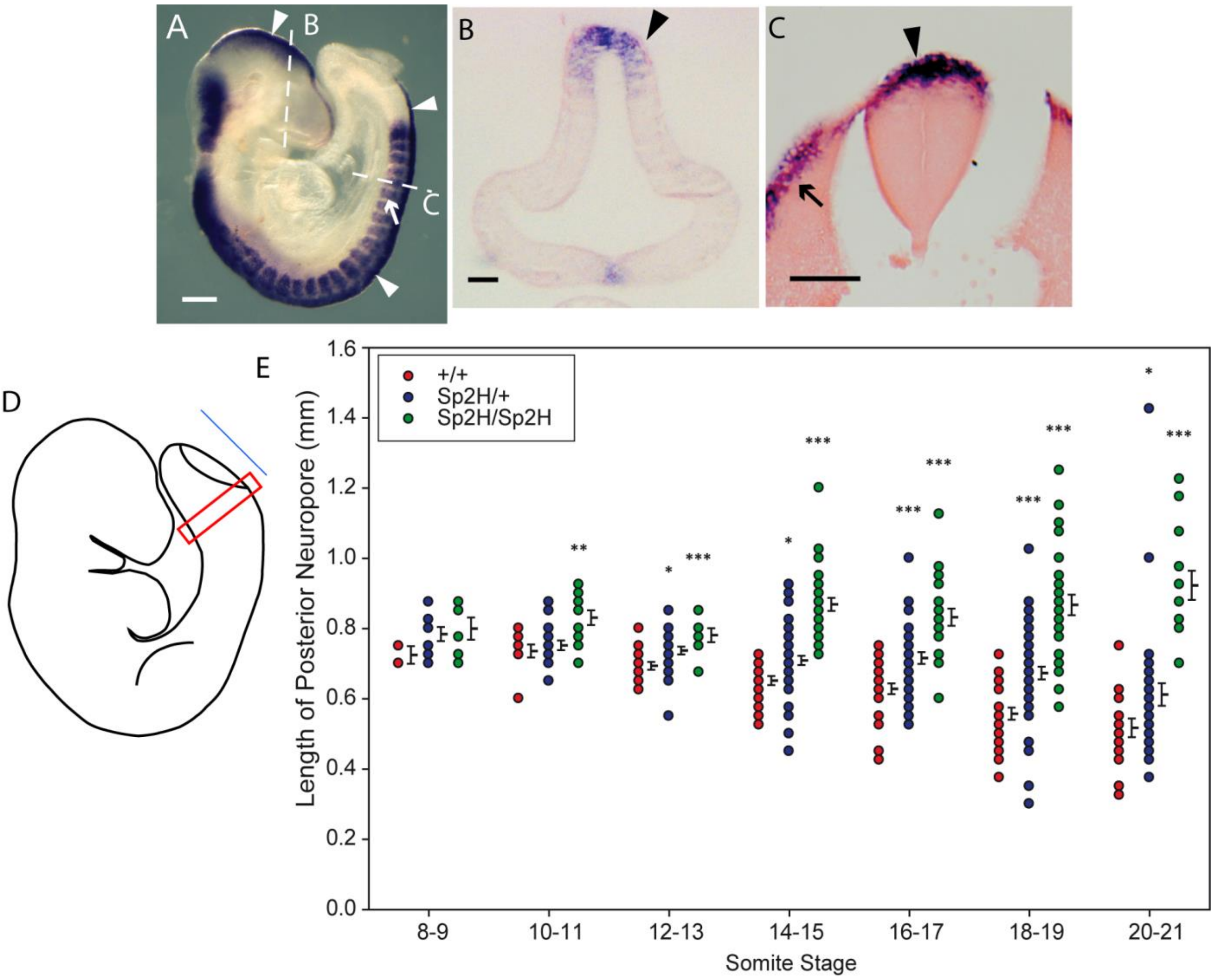

Figure S2. *Pax3* expression in the dorsal neural epithelium and failure PNP closure in *Pax3*$^{Sp2H/Sp2H}$ mutant embryos. (A-C) *In situ* hybridisation for *Pax3* shows expression in the dorsal neuroepithlium (arrowheads) in the cranial (B) and spinal (C) regions, as well as dermomyotome (arrow). (D-E) Raw data corresponding to Fig. 3A. Measurements of PNP length (indicated by blue line in D) show enlargement of PNP length in *Pax3*$^{Sp2H/2H}$ embryos from E9 (10-11 somite stage) onwards. Delayed PNP closure is also evident in *Pax3*$^{Sp2H/+}$ embryos compared with stage-matched +/+ controls (*p<0.05; **p<0.01; ***p<0.001; ANOVA).

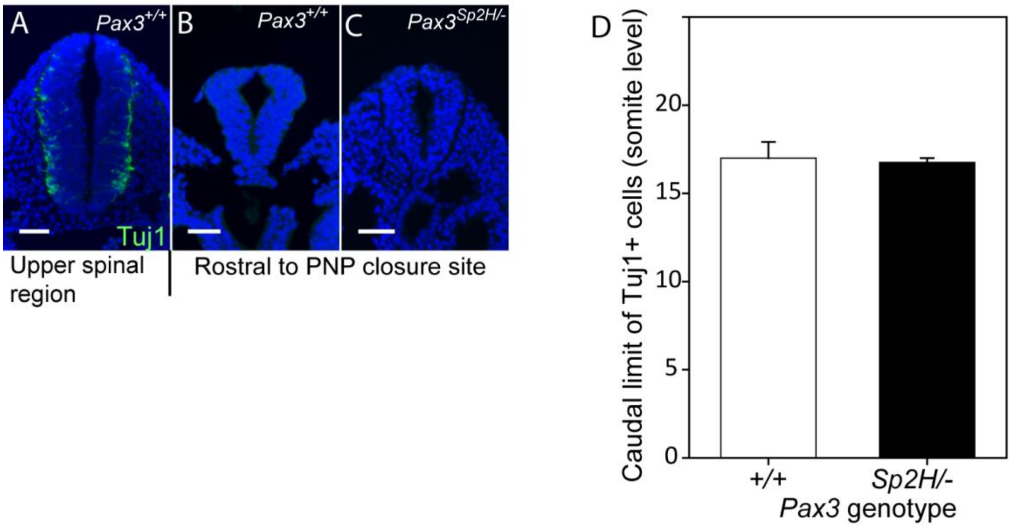

Figure S3. Neuronal differentiation does not occur at the level of PNP closure in wild-type or *Pax3* mutant embryos (related to Fig. 4). (A-C) Immunostaining for βIII-tubulin (Tuj1 in embryos at E9.5 (17-21 somite stage) confirms presence of positive cells at upper spinal levels (A) as seen in whole mount staining (Fig. 4B-C), whereas no positive cells are present the level of the recently closed neural tube rostral to the PNP closure site (B, C) in either wild-type or *Pax3* mutant embryos (images are representative of 3 embryos of each genotype; scale bar represents 50 µm). (D) The caudal-most limit at which βIII-tubulin (Tuj1) positive cells were detected was defined on the basis of the level of the somite adjacent to the neural tube. Among embryos at late E9.5 (23-24 somite stage), there was no significiant difference in the caudal level to which neuronal differentiation had progressed (n = 4 embryos per genotype).

Disease Models & Mechanisms • Supplementary information

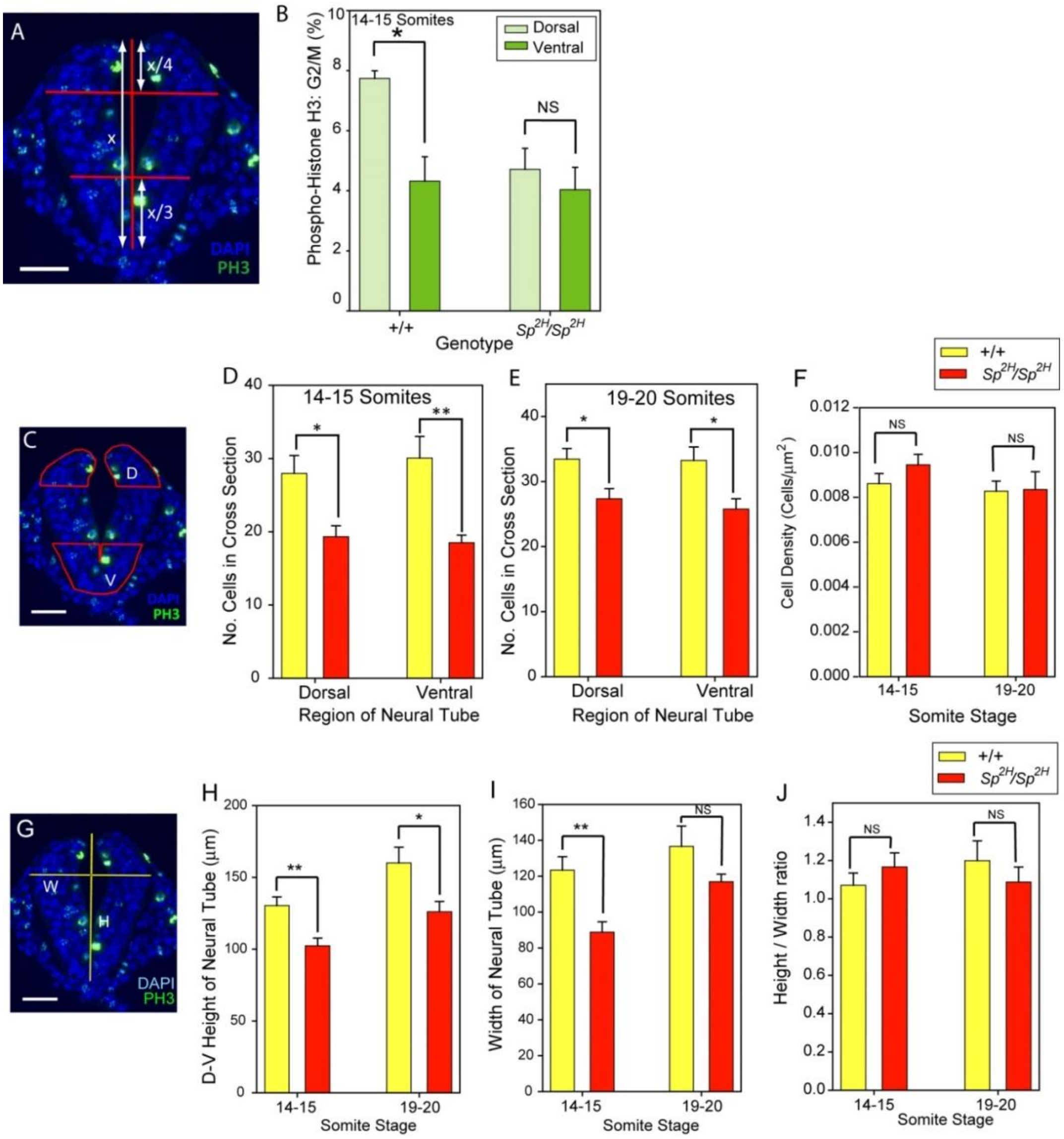

Figure S4. Analysis of proliferation, cell numbers and spinal neural tube size among wild-type and *Pax3*$^{Sp2H/Sp2H}$ mutant embryos at E9-9.5. (A-B) Immunostaining for PHH3 shows a significant dorsal-ventral difference in the number of cells at late G2-M phase in wild-type embryos (*P<0.05) but not in *Pax3* mutants (data re-plotted from Fig. 3). Dorsal and ventral regions used for analysis (A) correspond to Pax3-positive and negative regions, respectively. (C-E) The number of cells in transverse sections through the spinal neuroepithelium was lower in *Pax3*^{Sp2H} mutants than in +/+ embryos in both the dorsal and ventral regions (labelled D and V in panel C) at the 14-15 and 19-20 somite stages. (F) Overall cell density (cells/mm^2) did not differ between genotypes. (G-J) The dorsal-ventral height (H) and lateral width (W) of the spinal neuroepithelium was diminished in *Pax3*^{Sp2H} mutant embryos, but the height/width ratio did not differ from wild-type. (*P<0.05; **P<0.01; ANOVA); scale bars represent 50 µm. Bars represent mean ± SEM from 6 embryos per group (genotype and stage) with 5-6 sections per embryo (each bar corresponds to 32-36 sections).

Disease Models & Mechanisms • Supplementary information
